# Common carotid artery diameter responds to intravenous volume expansion: an ultrasound observation

**DOI:** 10.1186/s40064-016-2595-5

**Published:** 2016-06-23

**Authors:** Tobias Hilbert, Sven Klaschik, Richard K. Ellerkmann, Christian Putensen, Marcus Thudium

**Affiliations:** Department of Anesthesiology and Intensive Care Medicine, University Hospital Bonn, Sigmund-Freud-Str. 25, 53105 Bonn, Germany

**Keywords:** Common carotid artery, Sonography, Ultrasound, Diameter, Pulse pressure variation, Volume responsiveness, Cardiac surgery

## Abstract

**Background:**

In case of intravascular fluid depletion, large veins react to volume expansion with dilation. Little is known about the reaction of arterial vessels. We herein report on the effect of a standardized fluid bolus on the diameter of the common carotid artery (CCA) and its association with hemodynamic parameters, assessed in 20 mechanically ventilated patients after cardiac surgery. CCA was visualized using ultrasound, and the percentage increase in diastolic diameter was calculated by measuring before and after administration of crystalloid infusion solution. Invasive arterial blood pressure and pulse pressure variation (PPV) were assessed in parallel.

**Results:**

Median diastolic CCA diameter was 6.2 (Q_1_–Q_3_: 5.4–7.1) mm, and it significantly increased to 6.7 (5.8–7.3) mm upon fluid administration [5.0 (1.9–10.5) % increase]. Mean arterial blood (MAP) pressure likewise increased from 68 (70–73) to 85 (71–100) mmHg, whereas PPV was significantly reduced from 17.6 (16.8–23.9) to 13.2 (6.7–18.1) %. There was a significant association between the change in CCA diameter and the hemodynamic response (delta-MAP: r = 0.53, delta-PPV: r = 0.56; p < 0.05). Furthermore, carotid diameter measured before volume expansion significantly correlated with the delta-PPV upon fluid administration (r = −0.5; p = 0.02).

**Conclusions:**

Diameter of the CCA increases in response to intravascular volume expansion. Additional studies on the interplay between carotid geometry and intravascular fluid status are necessary.

## Background

In recent years, bedside ultrasound has become an important tool for the simple and non-invasive hemodynamic assessment of critically ill patients (Schmidt et al. 2012). This applies not only to echocardiography but also to ultrasound of large extra-thoracic veins (internal jugular vein, inferior vena cava) (Wesson et al. 2015; Guarracino et al. 2014; Zhang et al. 2014). In case of intravascular fluid depletion, venous vessels react to volume expansion with an increase in diameter that can be demonstrated by ultrasound. The extent of dilation is associated with changing hemodynamic variables such as cardiac output (CO) (Guarracino et al. 2014; Caille et al. 2008). However, little is known about the reaction of arterial vessels. Our main objective was to explore the effect of a standardized bolus of crystalloid infusion solution on the diameter of the common carotid artery (CCA) as an easily accessible arterial vessel in individuals supposed to be fluid demanding. We performed measurements in mechanically ventilated patients after cardiac surgery using bedside ultrasound. Hemodynamic parameters were assessed in parallel.

## Methods

This observation was conducted at the cardiac-surgery intensive care unit (ICU) of the University Hospital Bonn, Germany, after approval by the Institutional Review Board (IRB; protocol no. 206/14; date of approval August 13, 2014). In accordance with the judgment of the IRB, all measurements were considered part of routine practice and so, informed consent was waived. All patients age more than 18 years arriving at the ICU after elective cardiac surgery using heart–lung machine were screened for the following exclusion criteria:

- Atherosclerotic plaques or stenosis of the assessed carotid artery
- History of carotid surgery
- Thorax left open upon completion of surgery
- Cardiac arrhythmia (e.g., atrial fibrillation)
- History of radiotherapy or surgery of neck region
- Bilaterally inserted venous catheters (jugular or subclavian vein)
- Spontaneous breathing activity

All patients were clinically assessed by a blinded ICU consultant. Only patients being supposed to be volume demanding [based on clinical signs of inadequate tissue perfusion (e.g., escalating vasopressor requirement, decreasing urine output, etc.)] were included into the observation. At the ICU, patients were sedated using propofol and sufentanil and controlled mechanically ventilated [airway pressure release ventilation (APRV), V_T_ 8 ml/kg BW, P_peak_ ≤ 25 cm H_2_O, 10 cm H_2_O ≤ positive end-expiratory pressure (PEEP) ≤ 13 cm H_2_O, average I:E ratio 1:1.5 (individual titration to ensure complete expiration and avoid intrinsic PEEP), S_a_O_2_ ≥ 95 %, P_a_CO_2_ ≤ 45 mmHg]. Sonographic measurement of the CCA was performed using a Philips HD15 ultrasound device (Philips Healthcare, Hamburg, Germany), equipped with a linear transducer (L12-3 Broadband Linear Array Transducer, Philips Healthcare). By default, patients arriving at the ICU after cardiac surgery had received a central venous catheter preoperatively, usually inserted into the right internal jugular vein. To avoid any risk of infection at the puncture site, sonographic measurements were conducted on the contralateral side only, usually on the left. All sonographic images were obtained by two board-certified anesthesiologists, each with experience of more than 100 ultrasound-guided central venous cannulations. The hemodynamic data were collected by another investigator, blinded for the results of sonography. Ultrasound examinations and collection of hemodynamic data were performed with the patient in supine position with the head of bed 30° elevated (semi-recumbent). CCA was visualized by placing the ultrasound transducer perpendicular to the skin in a transverse plane on the patient’s neck lateral to the cricoid cartilage (short axis view). The artery was identified by compression as well as by color Doppler imaging. To avoid any influence of external compression on the CCA diameter during the following examination, sufficient ultrasound gel was used to allow the transducer to lose direct skin contact, thus applying the least possible amount of pressure. A B-Mode scan was recorded simultaneously with the electrocardiogram, the image was frozen at end-expiration, and the diastolic antero-posterior diameter of the CCA was measured in centimeters from intimal to intimal edge within the frozen B-Mode image. The procedure was repeated two times, and the mean diastolic CCA diameter was calculated from these three measurements. Thereupon, the transducer was removed and its location was marked on the skin.

Systolic, diastolic and mean arterial pressure (MAP) and pulse pressure variation (PPV) were collected using a Dräger Infinity C700™ monitor (Dräger Medical GmbH, Lübeck, Germany) via indwelling central venous and arterial catheters (left radial artery) as ‘snapshot’ measurements. The pressure transducer was adjusted to the level of the patient’s right atrium. In addition to ultrasound examination and hemodynamic data, other patient data recorded included age, sex, height, weight, Simplified Acute Physiology Score (SAPS) II, procedure, ventilation settings and vasoactive drug infusion rates (Le Gall et al. 1993).

After the initial assessment, a crystalloid infusion solution (Jonosteril™ 1/1, Fresenius Kabi, Germany) was administered (30 ml/min). Immediately after a total infusion volume of 7 ml/kg BW was reached, ultrasound measurement was repeated. Between these sequential measurements, neither patient position, nor ventilation parameters, nor infusion rate of vasoactive drugs were changed. The percentage increase in CCA diameter was calculated using the formula: [(diam_post_/diam_pre_) × 100] − 100. Furthermore, delta-MAP, defined as increase in MAP upon fluid expansion, and delta-PPV, defined as reduction of PPV, were calculated.

Statistical analysis was performed using GraphPad PRISM 5 (La Jolla, CA, USA). The coefficient of variation (CV) of measurements was calculated to illustrate the intra- and inter-observer reliability. Data are presented as median with percentile 25–75. Data from individual patients under varying conditions were compared using Wilcoxon matched pairs test. Spearman’s rank correlation coefficient was calculated to examine associations between ultrasound measurements and hemodynamic data. p values <0.05 were considered statistically significant.

## Results

Data from 20 consecutive patients were analyzed. According to routinely recorded SAPS II, patients showed mild organ dysfunction when arriving at ICU after cardiac surgery (median SAPS II = 26) (Le Gall et al. 1993). The need for post-operative vasoactive or inotropic support was moderate. An overview of the basic patients’ characteristics is given in Table 1. Under the sedation regime used, no patient showed spontaneous breathing activity.

**Table 1:** Basic patients characteristics

Age (years)	Median (range)	66 (52–78)
Male sex	*n* (percentage)	15 (75 %)
SAPS II	Median (range)	26 (18–34)
Administration of norepinephrine	*n* (percentage)	16 (80 %)
Infusion rate (µg/kg BW * min)	Median (Q_1_–Q_3_)	0.09 (0.04–0.12)
Administration of dobutamine	*n* (percentage)	18 (90 %)
Infusion rate (µg/kg BW * min)	Median (Q_1_–Q_3_)	3.55 (2.39–4.55)
Administration of milrinone	*n* (percentage)	1 (5 %)
Infusion rate (µg/kg BW * min)		0.21
Procedure	n (percentage)	
CABG		14 (70 %)
AVR		3 (15 %)
MVR		1 (5 %)
Comb. CAGB/VS		1 (5 %)
Myxoma		1 (5 %)
Total		20

*SAPS II* Simplified Acute Physiology Score II, *Q*_*1*_ *–Q*_*3*_ percentile 25–75, *CABG* coronary artery bypass graft, *AVR* aortic valve replacement, *MVR* mitral valve replacement, *Comb. CABG/VS* combined CAGB and valve surgery

**Fig. 1:**
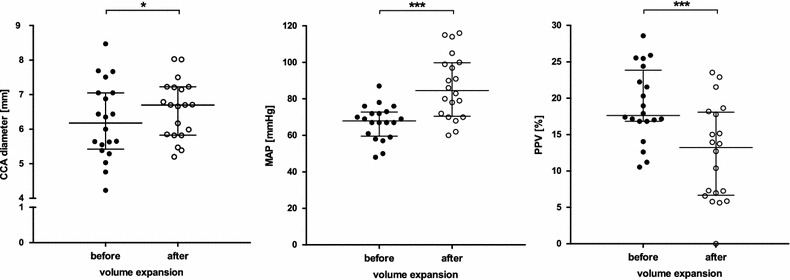
Effect of intravascular volume expansion on CCA geometry, MAP and PPV. CCA diameter increases with intravenous administration of crystalloid infusion solution (7 ml/kg BW) (*left*). MAP increases with intravenous administration of infusion solution (*middle*). PPV is reduced upon intravenous administration of fluid (*right*). *CCA* common carotid artery, *MAP* mean arterial pressure, *PPV* pulse pressure variation. Data are given as median with percentile 25–75. Wilcoxon matched pairs test, *p < 0.05; ***p < 0.001. *n* = 20

CCA was visible and easily identifiable in all patients. The percentage CV values, revealing high intra- as well as inter-observer reliability, were as follows: assessment 1: 12.93; assessment 2: 14.37; observer 1: 14.4; observer 2: 12.91. Median diastolic CCA diameter measured from intima to intima was 6.2 (5.4–7.1) mm prior to volume expansion (Fig. 1). Hemodynamic assessment revealed median values for MAP and PPV of 68 (60–73) mmHg and 17.6 (16.8–23.9) %, respectively. Immediately following administration of a bolus of crystalloid infusion solution (7 ml/kg BW), sonography was repeated and revealed a significant increase in median diastolic CCA diameter to 6.7 (5.8–7.3) mm (*p* = 0.03). Overall CCA diameters increased by 5.0 (1.9–10.5) % in comparison to the arterial diameters measured prior to fluid bolus in all patients. MAP likewise increased upon expansion of intravascular volume [85 (71–100) mmHg, *p* < 0.001], whereas PPV was significantly reduced to 13.2 (6.7–18.1) % (*p* < 0.001).

**Fig. 2:**
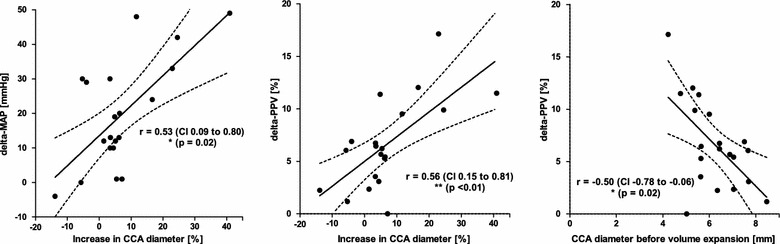
Association between CCA geometry, MAP, and PPV before and after intravascular volume expansion. *Left* *Scatter plot* showing the relation between the increase in carotid diameter and the increase in MAP (delta-MAP) upon intravenous administration of crystalloid infusion solution (7 ml/kg BW). *Middle* *Scatter plot* showing the relation between the increase in carotid diameter and the reduction of PPV (delta-PPV) upon intravenous administration of infusion solution. *Right Scatter plot* showing the relation between carotid diameter measured before intravascular volume expansion and the reduction of PPV (delta-PPV) upon intravenous administration of infusion solution. *CCA* common carotid artery, *MAP* mean arterial pressure, *PPV* pulse pressure variation. *Curved lines* indicate confidence band, *r* = Spearman’s rank correlation coefficient, CI = 95 % confidence interval. *p < 0.05; **p < 0.01. *n* = 20

Spearman’s rank correlation analysis revealed that the increase in CCA diameter following fluid administration was significantly associated with an increase in MAP (delta-MAP; Fig. 2) as well as with the reduction of PPV (delta-PPV). Interestingly, individuals with minimal hemodynamic response to fluid administration demonstrated an even decreasing CCA diameter. On the other hand, there was a significant negative correlation between carotid diameter measured prior to volume expansion and the delta-PPV upon fluid administration. Neither carotid diameter nor MAP nor PPV before or after administration of fluid were associated with the patients’ body mass index (BMI; not shown).

## Discussion

The main finding of the herein presented observation is that the diameter of the CCA measured using bedside ultrasound responds to intravenous fluid expansion with significant dilation. This was assessed in mechanically ventilated, critically ill patients. The extent of arterial dilation significantly correlated with increasing blood pressure and a reduction of PPV. To our knowledge, this is the first report about an association between sonographically assessed carotid artery diameter and dynamic variables describing intravascular volume status.

Expansion of the intravascular volume by means of intravenous administration of fluid is commonly performed in case of hypotension. Upon entering the vasculature, administered fluids are spatially distributed throughout the vascular bed. Approximately 65–85 % of the overall blood volume is pooled inside venous capacitance vessels, which thus serve as the reservoir of circulation (Klabunde 2012). In case of intravascular volume depletion (e.g., due to maldistribution or hemorrhage), the lack of venous filling may lead to decreased tension of the vascular wall, thus inducing changes in vessel geometry (collaps). On the other hand, dilated venous vessels may be indicative of proper intravascular filling. In recent years, these findings have been implemented into clinical diagnostic routine, as the sonographic assessment of the diameter of large veins helps to deduce the intravascular volume status of critically ill patients (Marik 2009).

In contrast to the veins, the relative bold muscular layer of the arterial wall prevents vessel collapse and allows the arteries to adjust their diameter in response to differing hemodynamic conditions. Reacting to changes in blood pressure and the chemical composition of the plasma, the autonomic nerve system regulates the arterial vascular tone via adrenoceptor stimulation (Klabunde 2012). In addition, local paracrine mechanisms, comprising, i.a., adenosine derivatives, nitric oxide (NO), and hormones such as endothelins, prostaglandins, and histamine, affect arterial wall tension (Storkebaum and Carmeliet 2011). At last, peripheral systemic resistance is regulated in response to the flow of blood itself (Bruno et al. 2014). These mechanisms serve to prevent organ damage, since blood is re-distributed from bradytrophic to tissues with high metabolic activity in case of hemodynamic instability. In summary, the autonomously controlled vascular tone regulates vessel geometry and thus blood pressure on the arterial side, which is in contrast to the veins that are rather mechanically dilated by the intrinsic pressure of increasing intravascular volume, e.g., in case of fluid administration (Marik 2009).

We administered a standardized bolus of crystalloid infusion solution within about 15 min and noted a subsequent significant increase of CCA diameter measured during cardiac diastole (Fig. 1). This corresponds to what Marik et al. described when using carotid artery Doppler ultrasound to assess hemodynamically unstable patients (Marik et al. 2013). In that study, a passive leg raising (PLR) maneuver, used as a temporary and reversible volume loading, induced an 80 % increase in carotid blood flow. Interestingly, similar to our observation, the simultaneously assessed diameter of the CCA likewise increased in that study. However, according to the formula “flow rate = ¼ × π × diameter^2^ × velocity”, an, e.g., 20 % increase in diameter contributes to more than the half of the increase in carotid blood flow (Nocke et al. 2014).

The global change in arterial diameter following fluid administration we observed correlated with increasing MAP and a reduction of PPV (Fig. 2, left and middle panel). High values for PPV have been demonstrated to be indicative of fluid responsiveness in mechanically ventilated patients (Perel et al. 2014). Interestingly, we furthermore noted a significant correlation between the reduction of PPV (delta-PPV) and the CCA diameter measured before administration of fluid (Fig. 2, right panel), independent of the patients’ weight and height. We found that the smaller the initial carotid diameter was, the more pronounced the patient reacted to expansion of intravascular volume. Several authors have shown that the increase in CO seen in volume demanding patients following fluid substitution likewise corresponds to the delta-PPV (Le Manach et al. 2012; Michard et al. 2000). To our knowledge, a relation between CCA diameter and changing PPV has not yet been reported so far and should be further investigated, maybe using invasive CO monitoring in a larger and more heterogeneous patient population.

PLR, as utilized by Marik et al., as well as intravascular volume expansion both reduce PPV in fluid demanding patients (Geerts et al. 2011). Furthermore, it has been shown that the diameter of the brachial artery increases in response to a PLR maneuver (Bapat et al. 2013; London et al. 1990). This ‘flow mediated dilation’ is described to be caused by endothelial shear stress, leading to increased NO syntheses as well as a reduction of endothelin secretion (Bruno et al. 2014; Morawietz et al. 2000; Lüscher and Barton 1997). In addition to such local, paracrine regulation, baroreflex activation is involved in arterial diameter regulation (Girerd et al. 1989). Thus, our observation of significant CCA dilation after volume expansion as well as the results described by Marik et al. can well be explained by one or both of these physiological mechanisms.

Our report generates hypotheses, but it certainly has limitations that deserve a critical discussion. First, due to the lack of direct assessment of CO, the observed association of CCA diameter with delta-PPV does not necessarily demonstrate a relationship between CCA diameter before volume loading and a variation in CO. Of course, additional studies using extended invasive hemodynamic monitoring are necessary to analyze the relation between carotid geometry and intravascular fluid status. Second, the observation described herein was performed in a small (yet homogenous) population, revealing significant associations using non-parametric tests. Of course, future studies should be performed on a larger collective. Third, repeated sonographic measurement of CCA diameter was performed by the same investigator as the initial assessment and was thus not truly blinded in this aspect. Last, although carotid diameter was not associated with the patients’ body dimensions, due to the close dependency of CCA diameter and delta-PPV, it may be hard to rule out any patient-related confounding factors on the CCA diameter measurement.

## Conclusions

Our results suggest that the diameter of the CCA responds to intravascular volume expansion with significant dilation in patients clinically appearing fluid demanding. Additional studies including invasive hemodynamic monitoring should shed more light on the interplay between carotid geometry and intravascular fluid status.

## Abbreviations

CO: cardiac output
CCA: common carotid artery
ICU: intensive care unit
IRB: institutional review board
APRV: airway pressure release ventilation
V_T_: tidal volume
BW: body weight
P_peak_: peak inspiratory pressure
PEEP: positive end-expiratory pressure
I:E ratio: inspiration to expiration ratio
S_a_O_2_: arterial oxygen saturation
P_a_CO_2_: partial pressure of carbon dioxide in arterial blood
MAP: mean arterial blood pressure
PPV: pulse pressure variation
SAPS II: Simplified Acute Physiology Score II
CV: coefficient of variation
BMI: body mass index
NO: nitric oxide
PLR: passive leg raising
SVV: stroke volume variation
